# P-879. Predicting Antimicrobial Resistance and Empiric Treatment Failure in Hospital-Onset Sepsis Using Machine Learning on Electronic Health Records

**DOI:** 10.1093/ofid/ofae631.1070

**Published:** 2025-01-29

**Authors:** Scott A Cohen, John G Morris, Mattia Prosperi

**Affiliations:** University of Florida, Gainesville, Florida; University of Florida, Gainesville, Florida; University of Florida, Gainesville, Florida

## Abstract

**Background:**

Antimicrobial resistance (AMR) is a major cause of treatment failure in hospital-onset sepsis. Approaches to guide empiric antimicrobial therapy are urgently needed. We utilized machine learning to predict empiric treatment failure and AMR patterns within a high-risk hospital-onset sepsis cohort using electronic health records (EHRs).

**Methods:**

We examined hospitalizations with documented bacterial infection from 2010 to 2023 at a tertiary-care academic hospital system. Hospital-onset sepsis was defined using CDC Adult Sepsis Event criteria. Empiric therapy was considered adequate if all pathogens isolated were susceptible to antimicrobials administered. AMR patterns included methicillin (MRSA), extended-spectrum beta-lactamase (ESBL), vancomycin (VRE), ceftriaxone, and carbapenem (CRE). Features for prediction were patient characteristics, hospitalization details, and clinical severity metrics measured within 24 hours prior to sepsis onset. Linear and non-linear machine learning models were evaluated using bootstrap validation and out-of-bag area under the receiver operating characteristic curve (AUC).

**Results:**

Among 49,581 hospitalizations, 2019 (4%) hospital-onset sepsis encounters were identified. Mean (SD) age was 65 (16.4) years and 900 (45%) were female. At 24 hours after sepsis onset, 377 (19%) received inadequate empiric treatment and 911 (45%) infections expressed AMR. Those who received inadequate empiric treatment were 8.2 (95%CI: 6.2-10.9) times more likely to have an AMR infection. Random forest model was best-performing, with AUC (95%CI) for any AMR pattern, VRE, and CRE at 0.64 (0.63-0.64), 0.71 (0.69-0.72), and 0.70 (0.67-0.73), respectively. The AUC (95%CI) for predicting inadequate empiric antimicrobial treatment was 0.65 (0.64-0.66) with a sensitivity of 64% (58-69%) and specificity of 52% (46-68%) at optimal probability thresholds.

**Conclusion:**

In a hospital-onset sepsis cohort with prevalent AMR, machine learning approaches utilizing limited EHR data accessible at initial sepsis recognition had moderate, but clinically insufficient, discriminatory ability in predicting AMR and empiric antimicrobial treatment failure. Future research will evaluate the utilization of large language models using unstructured EHRs.

**Disclosures:**

**All Authors**: No reported disclosures

